# Left bundle branch area pacing for cardiac resynchronisation therapy

**DOI:** 10.1007/s12471-019-01322-y

**Published:** 2019-09-05

**Authors:** L. M. Rademakers

**Affiliations:** Department of Cardiology, Catharina Ziekenhuis, Eindhoven, The Netherlands

A 72-year-old male patient with complete left bundle branch block (LBBB) with a QRS width of 180 ms (Fig. 2a), left ventricular ejection fraction of 30% and New York Heart Association (NYHA) class III heart failure despite optimal medical therapy was scheduled for implantation of a cardiac resynchronisation therapy defibrillator. The right atrial lead was placed in the appendage, while the right ventricular (RV) shock lead was positioned in a septal position. His bundle pacing failed due to a high capture threshold. Subsequently the 4.1F lumenless 3830 (SelectSecure, Medtronic, Minneapolis, MN, USA) pacing lead was delivered using the C315HIS (Medtronic) sheath in the left bundle branch area by means of a technique described by Huang et al. ([1]; Fig. 1). The capture threshold was 0.8 V/0.5 ms. Stimulation of the left ventricular (LV) lead with a short atrioventricular (AV) delay (80 ms) resulted in a typical right bundle branch block with a QRS width of 110 ms. During sequential ‘bi-septal’ pacing (LV 35 ms before RV) with an optimal AV delay (120 ms), the QRS width could be further shortened to 95 ms (Fig. 2b). The patient responded well to therapy, both in NYHA class (II) and echocardiographic measures.

**Fig. 1:**
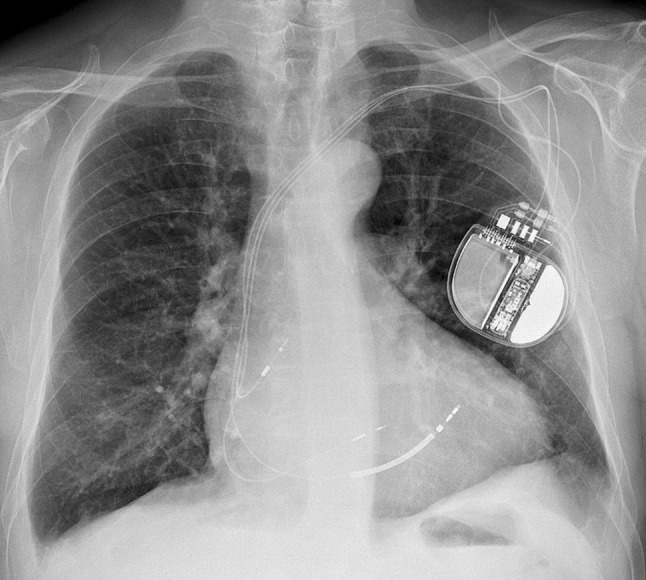
Chest radiograph in anterior-posterior projection demonstrating final position of right atrial lead, left ventricular lead and right ventricular shock lead

**Fig. 2:**
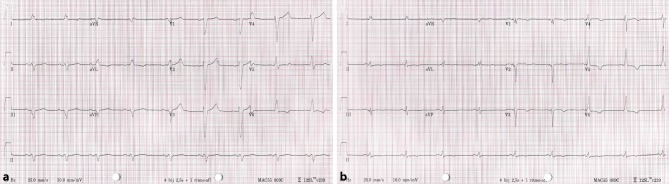
**a** Baseline electrocardiogram with complete left bundle branch block and wide QRS complex. **b** Electrocardiogram during sequential ‘bi-septal’ pacing with narrow QRS complex

## References

[1] Huang W, Su L, Wu S (2017). A novel pacing strategy with low and stable output: pacing the left bundle branch immediately beyond the conduction block. Can J Cardiol.

